# Supplementary material for: Circulating Trimethylamine-N-Oxide Is Elevated in Liver Transplant Recipients
Source: Int J Mol Sci. 2024 May 30;25(11):6031. doi: 10.3390/ijms25116031 (PMC11172608; doi:10.3390/ijms25116031)
Supplement: Supplementary file 1 [file ijms-25-06031-s001.zip › ijms-2993208-supplementary.pdf]

CIRCULATING TRIMETHYLAMINE-N-OXIDE AND MORTALITY IN LIVER TRANSPLANT RECIPIENTS

Maria Camila Trillos-Almanza[1*], Mateo Medina Chvatal[1], Margery A. Connelly[2], Han Moshage[1], TransplantLines Investigators, Stephan J. L. Bakker[3], Vincent E. de Meijer[4], Hans Blokzijl[1], Robin P.F. Dullaart[5].

SUPPLEMENTAL TABLES AND FIGURES

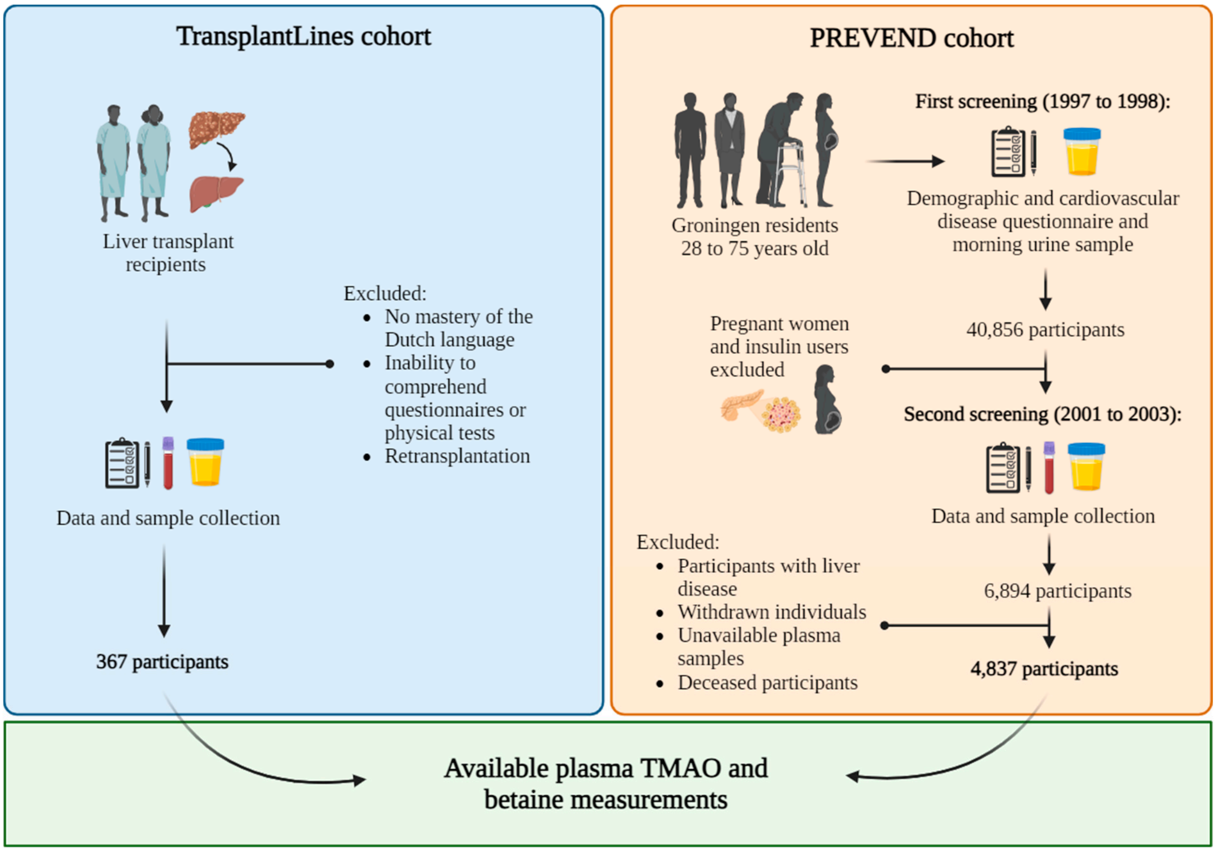

Supplemental Figure S1. Participant selection algorithm and cohorts descriptions.

The TransplantLines cohort comprises all liver transplant recipients at the University Medical Center Groningen until January 2021, who agreed to participate and met the inclusion

criteria. For the PREVEND cohort, invitations for the questionnaire and morning urine sample were extended to all Groningen residents aged 28 to 75, excluding pregnant women and insulin users. The current study included participants who successfully completed the second screening evaluation, did not report a liver disease and had TMAO and betaine measurements available. Figure created with Biorender.com

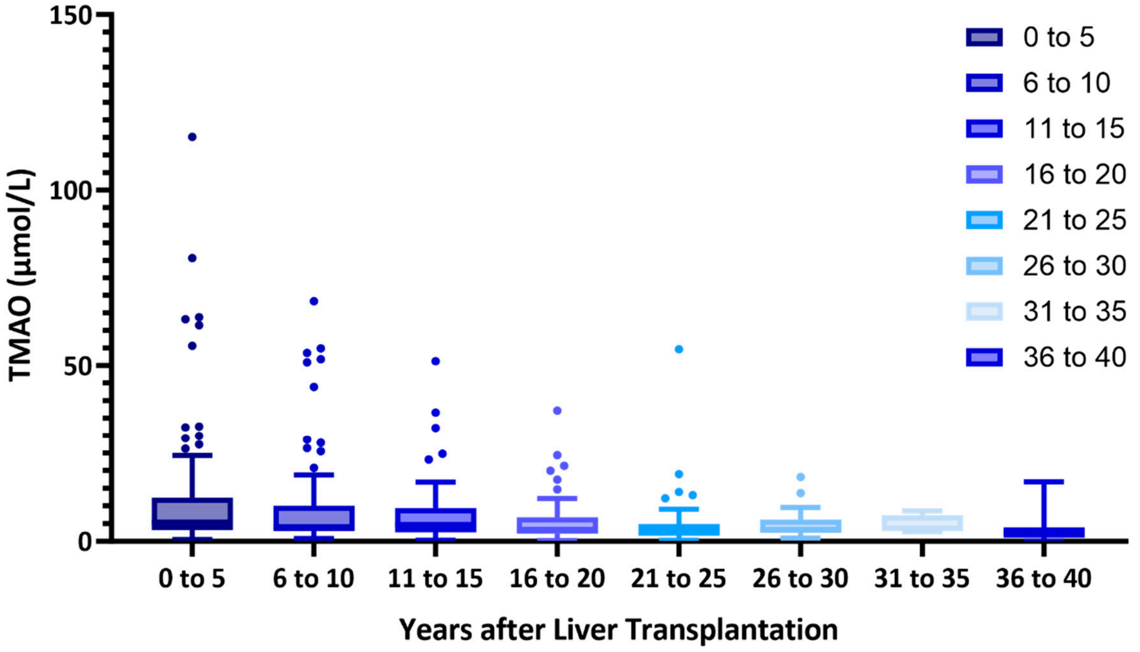

Supplemental Figure S2. TMAO distribution in LTR according to years after liver transplantation. Boxes represent the plasma TMAO concentrations (µmol/L), given as medians with interquartile ranges. Overall comparison with Kruskal-Wallis test, p = 0.0052.

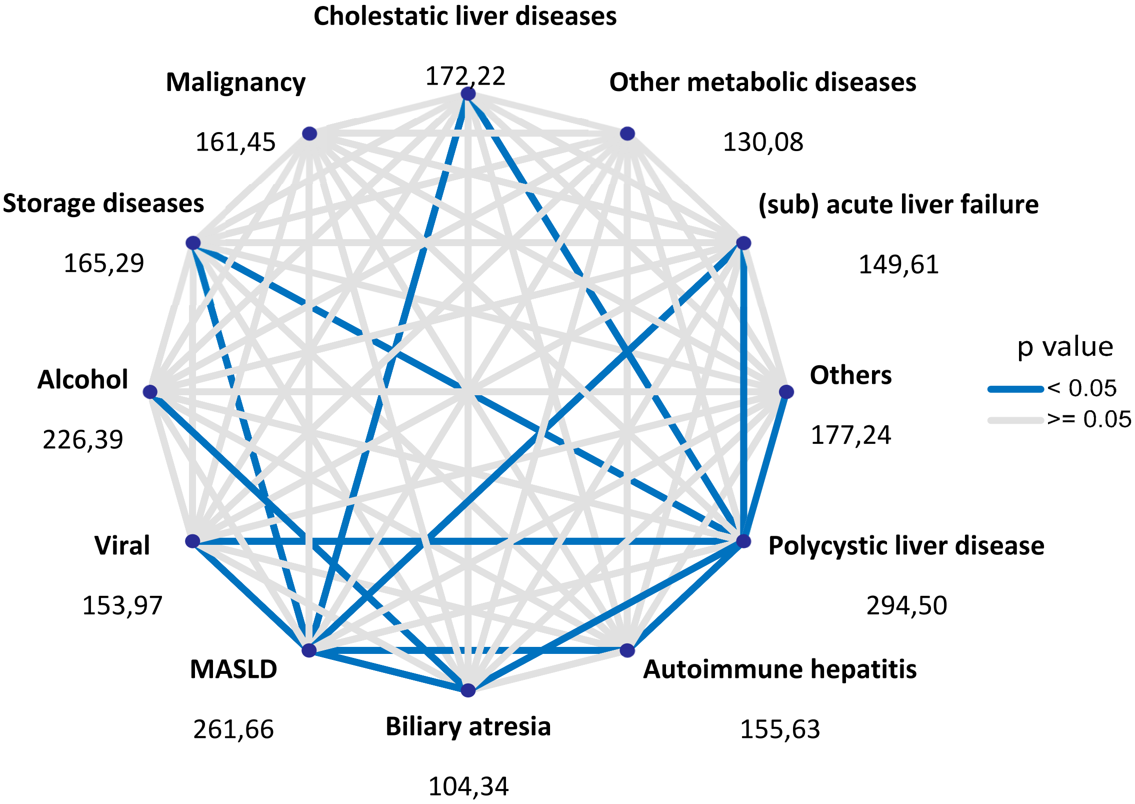

Supplemental Figure S3. Mean rank of plasma TMAO concentration according to the Kruskal-Wallis test.

Mean rank of plasma TMAO concentration across different etiologies, as determined by the Kruskal-Wallis test. Each node represents the average rank for a specific etiology group, and the connecting lines depict the distribution of values among these groups. Statistically significant differences are highlighted in blue, providing visual insight into variations in plasma TMAO concentration among the different etiologies.

Supplemental Table S1. Pearson correlation coefficients between the use of medication and TMAO after adjustment for confounders (see legends) in the liver transplant recipients.

	**TMAO (µmol/L) Adjusted for additional confounders**	
	Partial correlation coefficient	*p*-value
Antihypertensives	-0.052	.409
Glucose-lowering drugs	0.015	.818
Statins	-0.102	.107
PPI	-0.030	.640
Iron supplementation	**0.208**	**<.001**
Calcineurin inhibitors	-0.066	.295
Tacrolimus	-0.018	.774
Cyclosporin	-0.065	.305
Antimetabolite agents	-0.077	.224
Mycophenolate	-0.050	.430
Azathioprine	-0.039	.533
Glucocorticoids	-0.041	.520
Prednisolone	-0.064	.310
Prednisone	0.036	.570
Sirolimus	**0.127**	**.044**

Confounders were defined according to the univariate analysis in table 2; then the correlations were adjusted by age, sex, HbA1C, triglycerides, HDL cholesterol, and eGFR. TMAO and triglycerides were Ln transformed for correlation analysis. *Abbreviations: PPI: proton-pump inhibitors.*

Supplemental Table S2. Pearson correlation coefficients between clinical and laboratory variables and TMAO in the PREVEND cohort.

	**TMAO (µmol/L)**	
	Pearson correlation coefficients	*p*-value
Age	**0.123**	**<.001**
Sex	-0.013	.385
BMI	**0.038**	**.008**
Current smoking	**-0.033**	**.023**

Alcohol consumption	-0.010	.483
SBP	-0.001	.947
Fasting glucose	**0.068**	**<.001**
Total cholesterol	-0.009	.518
HDL cholesterol	-0.008	.595
Triglycerides	0.010	.470
eGFR	**-0.118**	**<.001**
Antihypertensives	0.002	.906
Glucose-lowering drugs	**0.054**	**<.001**
Statins	0.022	.120

TMAO and triglycerides were logarithmically transformed for correlation analysis. *Abbreviations: BMI, body-mass index; SBP, systolic blood pressure; HDL, high-density lipoproteins; eGFR, estimated glomerular filtration rate.*

Supplemental Table S3. Multivariable linear regression analyses demonstrating independent associations of circulating TMAO in PREVEND participants.

	**Model 1**		**Model 2**	
	Std. β (95% CI)	*p*-value	Std. β (95% CI)	*p*-value
Age	**0.730 (0.728, 0.732)**	**<0.001**	-0.033 (-0.036, -0.029)	0.105
BMI			-0.001 (-0.007, 0.006)	0.938
Current smoking			-0.030 (-0.095, 0.035)	0.054
Fasting glucose			**0.055 (0.025, 0.084)**	**0.001**
eGFR			**-0.129 (-0.131, -0.126)**	**<0.001**
Glucose-lowering drugs			0.027 (-0.143, 0.197)	0.096

Std. β: standardized regression coefficients. Model 1: adjusted for age. Model 2: Model 1 + BMI, current smoking, fasting glucose, eGFR and use of glucose-lowering drugs. *Abbreviations: BMI, body-mass index; eGFR, estimated glomerular filtration rate*

Supplemental Table S4. Association of TMAO per 1 Ln SD increment with all-cause mortality after liver transplantation in men vs women.

	TMAO per 1 Ln SD increment		T1	T2		T3	
**Men, n**	215		61	82		72	
Events, n	20		3	8		9	
**Women, n**	152		57	44		51	
Events, n	9		2	3		4	
	HR (95% CI)	*p*-value		HR (95% CI)	*p*-value	HR (95% CI)	*p*-value
Men							
Crude model	**1.03 (1.00, 1.06)**	**.037**	ref	3.03 (0.79, 11.55)	.105	**4.07 (1.09, 15.14)**	**.036**
Model 1	**1.03 (1.00, 1.07)**	**.046**	ref	2.35 (0.60, 9.15)	.218	2.78 (0.70, 10.95)	.145
Women							
Crude model	1.01 (0.96, 1.07)	.596	ref	1.97 (0.33, 11.79)	.458	3.98 (0.71, 22.18)	.115
Model 1	1.00 (0.95, 1.06)	.896	ref	1.51 (0.23, 10.07)	.673	2.90 (0.44, 19.06)	.269

Data are presented as hazard ratios with 95% confidence intervals and *p* values. Model 1: adjusted for age.

Supplemental Table S5. Distribution of all-cause mortality within the TMAO tertiles according to etiology.

Etiology	All-cause mortality (# events)			
	**Overall**	**T1**	**T2**	**T3**
Storage diseases	2	0	0	2
Autoimmune hepatitis	1	0	0	1
Cholestatic liver disease	6	2	2	2
Viral	3	0	2	1
ALD	5	1	2	2
MASLD	5	1	1	3
Malignancy	0	0	0	0
Biliary atresia	0	0	0	0
Acute and subacute liver failure	3	1	2	0
Polycystic liver disease	0	0	0	0
Others	4	0	2	2

Abbreviations: ALD, alcohol-associated liver disease; MASLD, Metabolic dysfunction associated steatotic liver disease.

Supplemental Table S6. Association of TMAO per 1 Ln SD increment with all-cause mortality after liver transplantation due to MASLD.

	TMAO per 1 Ln SD increment		T1	T2		T3	
Participants, n	**367**		**118**	**126**		**123**	
Events, n	**29**		**5**	**11**		**13**	
	HR (95% CI)	*p*-value		HR (95% CI)	*p*-value	HR (95% CI)	*p*-value
Crude model	**1.03 (1.00, 1.05)**	**0.023**	(ref)	2.50 (0.87, 7.21)	0.090	**4.14 (1.47, 11.66)**	**0.007**
Model 1	1.02 (0.99, 1.04)	0.093	(ref)	2.33 (0.80, 6.74)	0.120	2.67 (0.77, 9.28)	0.122

Data are presented as hazard ratios with 95% confidence intervals and p values. Model 1: crude model + MASLD.
